# Classification of Cocoa Beans by Analyzing Spectral Measurements Using Machine Learning and Genetic Algorithm

**DOI:** 10.3390/jimaging10010019

**Published:** 2024-01-08

**Authors:** Kacoutchy Jean Ayikpa, Pierre Gouton, Diarra Mamadou, Abou Bakary Ballo

**Affiliations:** 1 Laboratoire Imagerie et Vision Artificielle (ImViA), Université de Bourgogne, 21000 Dijon, France; kacoutchy-jean.ayikpa@u-bourgogne.fr (K.J.A.); mamadou.diarra1@ufhb.edu.ci (D.M.); bakary.abou@univ-fhb.edu.ci (A.B.B.); 2 Laboratoire de Mécanique et Information (LaMI), Université Felix Houphouët-Boigny, Abidjan 22 BP 801, Côte d’Ivoire

**Keywords:** spectral analysis, genetic algorithm, machine learning, spectral measurements

## Abstract

The quality of cocoa beans is crucial in influencing the taste, aroma, and texture of chocolate and consumer satisfaction. High-quality cocoa beans are valued on the international market, benefiting Ivorian producers. Our study uses advanced techniques to evaluate and classify cocoa beans by analyzing spectral measurements, integrating machine learning algorithms, and optimizing parameters through genetic algorithms. The results highlight the critical importance of parameter optimization for optimal performance. Logistic regression, support vector machines (SVM), and random forest algorithms demonstrate a consistent performance. XGBoost shows improvements in the second generation, followed by a slight decrease in the fifth. On the other hand, the performance of AdaBoost is not satisfactory in generations two and five. The results are presented on three levels: first, using all parameters reveals that logistic regression obtains the best performance with a precision of 83.78%. Then, the results of the parameters selected in the second generation still show the logistic regression with the best precision of 84.71%. Finally, the results of the parameters chosen in the second generation place random forest in the lead with a score of 74.12%.

## 1. Introduction

Ivory Coast is the world’s leading cocoa producer, with an annual production of around 2 million tons. This sector represents 15% of Ivorian GDP and 40% of export revenues. The quality of cocoa beans is a determining factor for the sector’s success. It influences chocolate’s taste, aroma, texture, and consumer satisfaction. Higher-quality cocoa beans are more valued internationally, allowing Ivorian producers to benefit from higher prices. The cocoa industry is a pillar of the Ivory Coast economy [1]. With this in mind, the Ivorian government and stakeholders in the cocoa industry are actively committed to improving the quality of cocoa beans. They invest in the research and development of innovative technologies aimed at increasing yield while providing producers with a deeper understanding of the quality of their products. These efforts help them make informed decisions to improve their agricultural practices [2]. In recent years, scientific research and technological innovation have converged to give rise to sophisticated systems capable of rapidly, accurately, and non-invasively analyzing colorimetric and spectral properties. This significant advancement has paved the way for creating artificial intelligence models that can assess the quality of cocoa beans in real time based on their unique visual and spectral characteristics [3,4].

As a result, using cutting-edge technologies, such as Spectral Analysis combined with Machine Learning and Optimization through the genetic algorithm, leads to a radical transformation of how we evaluate and classify cocoa beans [5].

The integration of artificial intelligence in this sector has marked a significant advancement, providing players in the cocoa industry, including producers, processors, and traders, with substantial benefits in terms of improved efficiency, precision, and profitability. Using a genetic algorithm as an optimization tool is a powerful method to refine and optimize the parameters that machine learning models use to achieve even better and more precise cocoa bean classification [6,7].

This study will explore the analysis of spectral measurements, machine learning, and the genetic algorithm to classify cocoa beans. This research represents a significant step in the search for advanced technological solutions to improve the quality and competitiveness of the cocoa industry while paving the way for innovation opportunities in agriculture and food processing.

Our contributions are as follows:

- Implementation of spectral measurements of cocoa beans: we have implemented a method to measure the spectral properties of three categories of cocoa beans. These properties are measured using a spectrometer, a device that measures the amount of light reflected by an object based on its wavelength.
- Classification of spectral measurements using a set of algorithms: we used a set of algorithms to classify the spectral measurements of the three categories of cocoa beans. These algorithms are based on machine learning, a discipline of artificial intelligence that allows computers to learn from data without being explicitly programmed.
- Selection of spectra using the best algorithm coupled with the genetic algorithm: we used the genetic algorithm to select the most relevant spectra for classification. The genetic algorithm is an optimization algorithm inspired by natural selection.
- Analysis and comparison of the different classifications: We analyzed and compared them to evaluate their performance.

## 2. Related Work

Extensive research has been undertaken in cocoa cultivation, as well as in the utilization of spectral measurements. Wei et al. developed a broad-spectrum detection method for OTA, OTB, and OTC to improve the accurate detection of ochratoxins in cocoa using colorimetric and spectral analysis [8]. However, gaps remain and deserve improvement. By expanding the sample size of cocoa beans, providing more detailed information about feature extraction methods, and evaluating the method’s performance on various varieties of cocoa beans, researchers could increase the accuracy and reliability of this approach. Lin et al. developed a new method to detect volatile markers in wheat infected with Aspergillus glaucus. The method combines a colorimetric sensor (CS) and visible/near-infrared spectroscopy (VNIR). The optimized Si-PLS model based on the HBDP sensor gave the best detection performance. The correlation coefficient of the prediction ensemble was 0.9387, indicating a strong correlation between the model predictions and the actual values [9]. However, the study used a dataset of 100 wheat samples, of which 50 were infected with Aspergillus glaucus and 50 were healthy. A larger sample size would be needed to confirm the study results. The study was conducted in a controlled culture chamber. The method’s performance could vary in different environments, such as agricultural fields. Hernández-Hernández et al. focused their study on the application of near-infrared (NIR) spectroscopy to efficiently identify promising cocoa genotypes based on their chemical composition, including levels of bioactive compounds, in more than 80 cocoa bean samples from the Mexican Germplasm Bank, collected over three years. Notably, different genotypes observed significant differences in chemical composition in fermented and dried cocoa samples. The research focused on determining the content of fat, protein, total sugars, total phenols, phenolic compounds, and theobromine in samples of whole cocoa beans and ground cotyledons. Calibration models were developed using the spectra of intact beans, raw cocoa beans, and ground cotyledons [10]. The study by Santika et al. looked into cocoa cultivation at Kebun Kalisepanjang Cocoa Center to assess the quality of cocoa beans. To achieve this objective, the study implemented the ANFIS method within an expert system, thus making it possible to consider the various external factors influencing quality. At the same time, to determine the quality of the cocoa beans, they used the genetic algorithm (GA), which played a key role in the evaluation process. The results obtained in this research are significant, with a root mean square error (RMSE) of 4.3, which testifies to the suitability of the algorithms used as an expert system for bean quality selection. This approach shows promise for improving the quality and management of cocoa production in this region [4]. Karadağ et al. undertook a study to identify healthy peppers from those infected with Fusarium wilt (Capsicum annuum). They used a spectroradiometer to analyze reflections coming from pepper leaves. These reflections were obtained from four groups of pepper leaves grown in a closed environment, including healthy peppers, peppers with fusarium wilt, peppers with the presence of mycorrhizal fungi, and peppers with fusarium wilt with mycorrhizal fungi. The measurements were carried out over a wavelength range from 350 nm to 2500 nm. The pepper disease detection process occurs in two distinct phases. In the first step, a feature vector is created using the coefficients from the wavelet decomposition and the statistical values of these coefficients. The second step is to classify the feature vectors of the input data. Three methods were used for this classification, namely artificial neural networks (ANN), Naive Bayes (NB), and the k-nearest neighbors (KNN) method. The results obtained are satisfactory [11]. Chen et al. carried out a study to identify sensitive spectral bands and develop a hyperspectral vegetation index specific to detecting leaf spots. The canopy hyperspectral reflectance spectra of leaf-spot-susceptible peanut varieties were measured at two experimental sites in 2017. The normalized difference spectral index (NDSI) was derived based on their correlation with the Disease index (DI) in the leaf spectrum between 325 nm and 1075 nm. The results showed that the spectral reflectance of the vegetation cover significantly decreased in the near-infrared (NIR) regions as the disease index increased (r < −0.90). The spectral index for detecting peanut leaf spots was LSI: (NDSI (R938, R761)), with R^2^ values reaching 0.68 for the regression model [12].

## 3. Materials and Methods

The experiments used Python programming at the ImViA laboratory, utilizing a DELL desktop computer. This computer has an Intel^®^ Core i7-10700 CPU (Dell, Montpellier, France) running at 2.90 GHz, 32 GB of RAM, and an NVIDIA Quadro P400 GPU (NVIDIA, Biot, France). The models were configured within Python version 3.8.8, utilizing Keras API version 2.4.3, TensorFlow version 2.3 as the backend, and CUDA/CuDNN dependencies for GPU acceleration.

The Konica-Minolta CS-2000 (Konica Minolta, Bourges, France) is an advanced optical spectroradiometer designed to accurately measure the visible light spectrum, recording the different wavelengths and their respective intensity levels. It can dissect light into its spectral components, thereby providing an in-depth analysis of the spectral composition of light [13]. This device was used as part of our study to create our dataset. This includes spectral and colorimetric measurements of three distinct categories of cocoa beans: Category 1—fermented beans, Category 2—low-grade fermented beans, and Category 3—unfermented beans. The CS-2000 has allowed us to obtain detailed information on the spectral composition of the light emanating from these different categories of cocoa beans, which is essential for our analysis and research work. 

### 3.1. Spectral Measurement of Cocoa Beans

Spectral measurements were performed over a wavelength range from 380 nm to 780 nm, with a resolution of 10 nm for each measurement. 

In our study, we have three categories of cocoa beans in this case:

- Category 1: these are superior quality fermented and dried cocoa beans;
- Category 2: these are fermented and dried cocoa beans of intermediate quality;
- Category 3: these are unfermented and dried cocoa beans.

**Figure 1:**
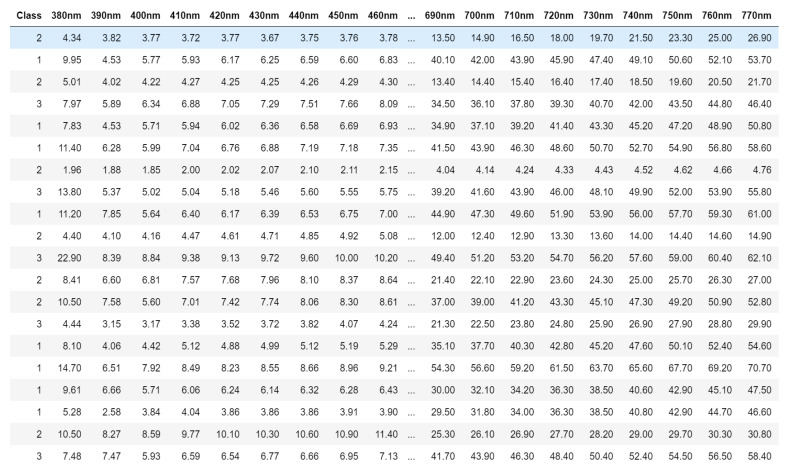
Extract of spectral measurement data from beans.

The cocoa beans used in our study come from several plantations selected with the assistance of agronomists in Côte d’Ivoire. Preparing the beans begins with harvesting the pods, carefully separating the beans and the pods, followed by the fermentation phase for 5 to 7 days in the shade, away from light, and then the drying process. The beans are sorted into three distinct categories and classified according to quality. Category 1 includes premium-quality fermented and dried cocoa beans, while Category 2 includes lower-quality fermented and dried cocoa beans after sorting. Category 3 concerns unfermented and dried cocoa beans. Currently, this sorting is carried out manually by growers, potentially leading to variations in quality. Figure 1 shows an extract of the data.

**Figure 2:**
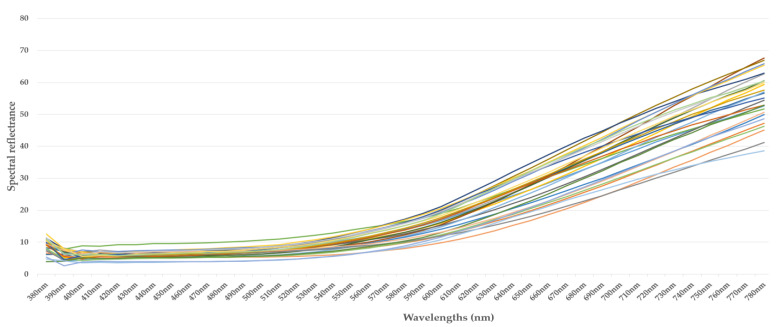
Curve of spectral reflectance data from cocoa beans—category 1. (each sample is represented by a different pseudo-color).

**Figure 3:**
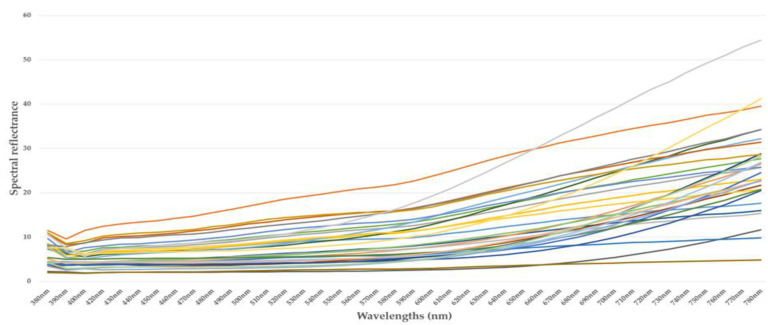
Curve of spectral reflectance data from cocoa beans—category 2. (each sample is represented by a different pseudo-color).

**Figure 4:**
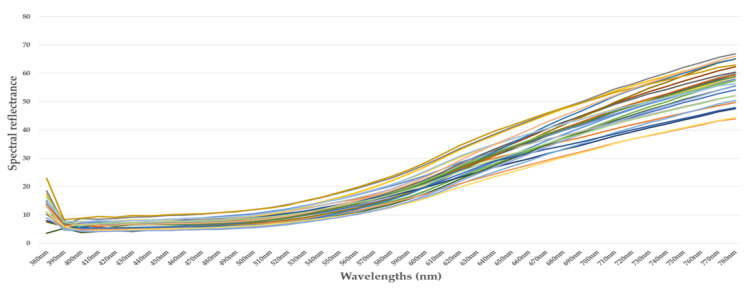
Curve of spectral reflectance data from cocoa beans—category 3. (each sample is represented by a different pseudo-color).

**Figure 5:**
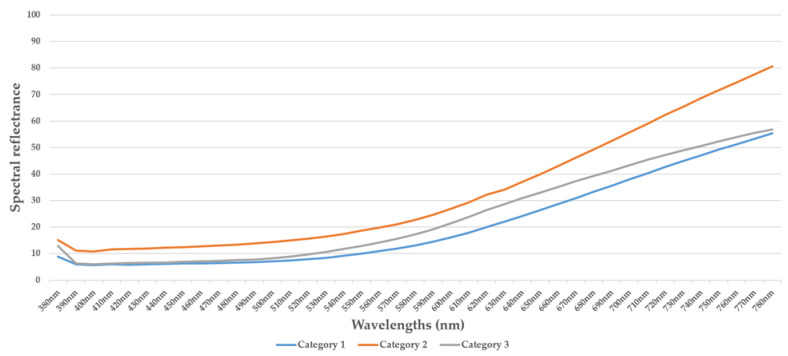
The average curve of spectral reflectance data for each category.

Figure 2, Figure 3, Figure 4 and Figure 5 illustrate the respective curves of each category of beans.

Figure 5 illustrates the curves representing the means of each category.

The measurements of the cocoa beans were carried out separately. The Minolta CS-2000 spectroradiometer was used for the measurements. This spectroradiometer is a precise machine that provides all the necessary information on the light produced by the source in the visible range (380–780 nm). The first step in our process is to measure the source response. We use a halogen source directed towards a Lambertian white for the total energy (Etotal). The second step involves measuring the reflectance of the cocoa bean on a non-reflective plate for total reflectance (ER).

**Figure 6:**
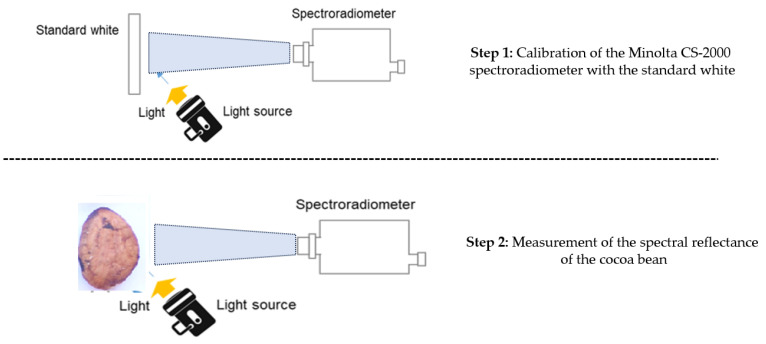
Protocol for spectral measurement of cocoa beans using the spectroradiometer.

The Reflectance (R) value is determined by calculating the ratio between the ER/Etotal, and the measurement is automatically provided by the device, thus establishing the relationship between the source’s response and the cocoa bean’s reflectance. The measurement setup is shown in Figure 6.

We separated our data, allocating 80% to the training and validation set while the remaining 20% were reserved for testing. We deliberately selected 30 cocoa beans from each category for our data collection. To carry out the spectral measurement of each bean, we used the CS2000 spectrometer. At the end of this procedure, we obtained a data set comprising 90 measures, each corresponding to a single bean. It is important to emphasize that, in our case, the possibility of increasing the data is limited since these are unique measurements for each bean. This constraint influences our analysis and modeling approach, requiring careful management of the available data to guarantee a faithful representation of the diversity of the spectral characteristics of the different categories of beans. In our measurement acquisition protocol, we take three measurements of a bean, and then we recover the average of these measurements, constituting the bean’s final measurement.

### 3.2. Machine Learning

Machine learning is a subfield of artificial intelligence that focuses on creating models and algorithms to learn from data and make predictions or classifications without explicit programming. In our work [14], we employed the following algorithms:

- SVM (Support Vector Machine): SVMs are used for classification and regression. They work by finding a hyperplane that maximizes the margin between classes in a multidimensional data space. They are effective for binary classification but can be extended to multi-class classification [15].
- Logistic Regression: Logistic regression is mainly used for binary classification and can be extended to multi-class classification. It models the probability that an observation belongs to a particular class. It uses a logistic function to estimate the probability based on the input features [16].
- Random Forest: Random forest is an ensemble algorithm that combines multiple decision trees. Each tree is trained on a random sample of the data, and the predictions are aggregated to obtain a final prediction. It is robust, less susceptible to overfitting, and effective for classification and regression [17].
- AdaBoost (Adaptive Boosting): AdaBoost is another ensemble algorithm that adjusts based on errors from previous models. It assigns different weights to observations based on their previous performance. It combines several weak models to create a robust model [18].
- Decision Tree: Decision trees are used for classification and regression. They divide the dataset into subgroups based on characteristics, using criteria such as entropy or Gini coefficient. They are easy to interpret but can suffer from overfitting [19].
- K-Nearest Neighbors (KNN): KNN is a similarity-based classification and regression algorithm. It assigns a class to an observation based on the classes of the k nearest neighbors in the feature space. It is simple to understand but can be sensitive to the distance used [20].
- XGBoost (Extreme Gradient Boosting): XGBoost is an improved implementation of gradient-boosted learning. It is efficient and effective, suitable for classification and regression. It uses regularization and advanced tree management to improve accuracy [21].

The choice of these models is motivated by the limited availability of spectral measurement data and their relevance for the analysis of tabular data. This selection is based on problem-specific considerations. SVMs are favored for their effectiveness in high-dimensionality nonlinear classification. Logistic regression is chosen for its simplicity and interpretability in linear tasks. Random forests are known for their robustness against overfitting and performance in complex datasets. AdaBoost improves weak models, while decision trees provide a graphical representation of decisions. KNN suits complex data structures, and XGBoost excels in exact nonlinear tasks.

### 3.3. Genetic Algorithm

Genetic algorithms (GAs) are a class of optimization algorithms inspired by the theory of Darwinian evolution. They are used to solve optimization and research problems in various fields, from system design to parameter optimization. Genetic algorithms are based on biological concepts such as natural selection, reproduction, and mutation.

Genetic algorithms are used in various fields, including parameter optimization, neural network design, planning, game strategy evolution, electronic circuit design, and many others. They are instrumental when the search space is ample, discontinuous, or complex to explore with traditional optimization methods [22,23,24].

The main components and stages of a genetic algorithm are as follows:

- Initial Population: An initial group of potential solutions is randomly generated. Each solution is represented as chromosomes or genotypes, which encode the parameters or characteristics of the solution.
- Evaluation: Each solution in the population is evaluated using an objective or fitness function. This function measures the quality of each solution to the optimization objective.
- Selection: Solutions are selected for reproduction based on their fitness. Higher-quality solutions are more likely to be chosen, thus simulating natural selection.
- Reproduction: Selected solutions are combined to create a new generation of solutions. This can include operations such as recombination (crossover) and mutation. Crossbreeding involves merging two parental solutions to produce offspring, while mutation slightly modifies one solution.
- Replacement: The new generation replaces the old generation, often using a fitness-based replacement model. This ensures that the best quality solutions are preserved.
- Stopping Criterion: The genetic algorithm runs for a certain number of iterations or until a predefined stopping criterion is reached or setting a maximum number of iterations.
- Result: The best solution found during the execution of the algorithm is returned as the optimization result.

The genetic algorithm plays a fundamental role in identifying the optimal combinations of parameters linked to spectral measurements in the visible range, covering from 380 nm to 780 nm. Thanks to evolution through generations, the genetic algorithm manages to develop effective combinations of different spectra, reducing the number of parameters. This simplification significantly contributes to facilitating the classification process. By tuning the parameters optimally, the genetic algorithm promotes a better understanding of spectral characteristics and thus improves the accuracy of data classification.

### 3.4. The General Architecture of the Methodology

Our comprehensive methodology includes the following steps:

1. Step 1: Spectral and Colorimetric Measurements

In this first step, we perform spectral and colorimetric measurements using the CS-2000 Optical Spectroradiometer. This spectroradiometer is a measuring device that quantifies the light reflected from a sample of cocoa beans. It generates a reflection spectrum representing the distribution of light intensities as a function of wavelength, generally covering the entire visible spectrum, from blue to red.

Spectral measurements, for their part, make it possible to characterize the spectral properties of cocoa beans by measuring the transmission of light at different wavelengths. This spectral information is essential for detecting subtle variations in the beans’ chemical composition, maturation, or quality.

2. Step 2: Data Export

Once the measurements are made, we export these data to standard format files, such as CSV (Comma-Separated Values). This step prepares the data for subsequent analysis.

3. Step 3: Data Division

We split the extracted data, creating three sets: training, validation, and testing data. This division is essential for the application of our models and algorithms. We separated our data, allocating 80% to the training and validation set while the remaining 20% were reserved for testing.

4. Step 4: Classification

In spectral measurements, we employ classification algorithms to categorize the obtained data. The algorithm that demonstrates the highest precision is selected for further analysis.

5. Step 5: Selection of the Best Spectral Parameters

In this last step, we refine our results using the algorithm selected in step 4, combined with genetic algorithms. Genetic algorithms help us identify and choose the best parameters for spectral measurements, thus allowing the realization of a new classification with all classification algorithms. The principle of the genetic algorithm is as follows:

a. First, we run a function to initialize a random population.
b. The randomized population is then subjected to the fitness function, which returns the best parents (with the highest accuracy).
c. The best parents will be selected according to the n-parents parameter.
d. After performing the same operation, the population will be subjected to the crossover and mutation functions, respectively.
e. The cross is created by combining the genes of the two most suitable parents by randomly selecting the first parent and part of the second parent.
f. The mutation is obtained by randomly inverting the bits selected for the child resulting from the crossover.
g. A new generation is created by selecting the most suitable parents from the previous generation and applying crossing over and mutation.
h. This process is repeated for five generations.

**Figure 7:**
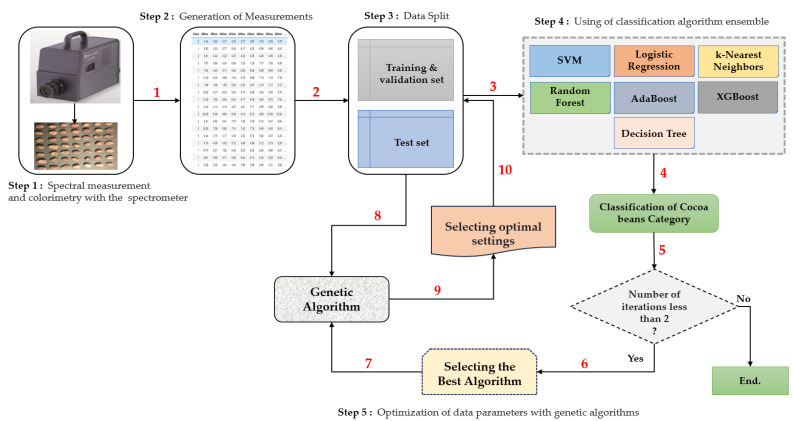
Schematic representation of our approach.

Figure 7 summarizes the structure of our methodological approach.

This comprehensive methodology allows us to deeply analyze the characteristics of cocoa beans, optimize our classification models, and obtain accurate and meaningful results for the classification of cocoa beans based on their spectral and colorimetric properties.

The functions and general algorithm present the essential operation and principle of the implemented model. The purpose of the Train_test_evaluate Algorithm 1 is to carry out the training and evaluation of the model, and is presented as follows:
**Algorithm 1** Train_test_evaluate**Input:** X_train, X_test, y_train, y_test, models **Output:** Score, Modelchoice **Begin** **Writing a function to train, test, and evaluate models:** **Creating the dataframe and initializing variables:**
acc_M = 0
Modelchoice = ””
Score = DataFrame({“Classifier”: classifiers})
  j = 0
**For each** model **in** the **models list**
             model = i
             **Training Phase:**
             Train Model (X_train, y_train)
             **Testing Phase:**
             predictions = model.predict (X_test)
             **Performance Evaluation:**
             **Metrics:** Accuracy, Precision, Recall, F1-score, Matthews Correlation Coefficient,
             Mean Squared Error: Acc, Preci, Re_Sc, F1_S,Mcc,Mse =
             *Classification_report*(y_test, predictions).
             **Saving model performance metrics:**
             Score[j,”Accuracy”] = Acc; Score[j,”f1_score”] = F1_S; Score[j,”recall_score”] = re_sc
             Score[j,”matthews_corrcoef”] = Mcc; Score[j, “precision_score”] = Preci; 
             Score[j, “Mse_score”] = Mse
             **Identifying the best model**
             **if** acc_M < acc **then**
                     acc_M = acc
                     Modelchoice = model
             **End**
           j = j + 1
      **Endfor**
      **return** Score, Modelchoice
**End Function**


Next, we expose the Genetic Algorithm 2, which implements the genetic algorithm.
**Algorithm 2** Genetic**Input: X, y, X_train, X_test, y_train, y_test, new_model** **Output:** Gene, score
**Begin**
**new_model = ML**
**Function initilization_of_population(size, n_feat)**
        **population** = Empty list
        **For** i **in range from** 1 **to** size
                **chromosome** = Boolean array of size **n_feat** initialized to **True**
                **For** j **in range from** 0 **to** int(0.3 * **n_feat**)                         **chromosome[j]** = False
                **Endfor**
                **Randomly** shuffle the elements in **chromosome**
                Append chromosome to the **population**
        **Endfor**
      **Return population**
**Endfunction**

**Function fitness_score (population, X_train, X_test, y_train, y_test)**
        **scores** = Empty list
        **For each** chromosome **in** population
                Create a model (**new**_**model**)
                Train the model on X_train using the features specified by the chromosome
                Predict class labels on X_test with the model
                Calculate accuracy score by comparing predictions with y_test
                Append the score to **scores**
          **Endfor**
        **Sort** scores in descending order while maintaining the correspondence with the population
        **Return** scores, sorted population
**Endfunction**

**Function selection (pop_after_fit, n_parents)**
        population_nextgen = Empty list
        **For** i **in range from** 1 to **n_parents**
                Append pop_after_fit[i] to population_nextgen
        **Endfor**
        **Return** population_nextgen
**Endfunction**

**Function crossover (pop_after_sel)**
        pop_nextgen = Copy pop_after_sel
        **For** i **in range from** 0 to the size of **pop_after_sel - 1 with a step of 2**
                new_par = Empty list
                child_1, child_2 = pop_nextgen[i], pop_nextgen[i+1]
                new_par = Concatenate the first half of child_1 and the second half of child_2.
                Append new_par to pop_nextgen
              **Endfor**
        **Return** pop_nextgen
**Endfunction**
**Function mutation (pop_after_cross, mutation_rate, n_feat)**
        mutation_range = Integer rounded down of mutation_rate times n_feat
        pop_next_gen = Empty list
        **For** each individual in pop_after_cross
                chromosome < = Copy the individual
                rand_posi < = Empty list
              **For** i **in range from** 1 to mutation_range
                        pos < = Random integer between 0 and n_feat - 1
                        Append pos to rand_posi
                **Endfor**
                **For each** position in rand_posi
                        Invert the value of the gene corresponding to that position in chromosome
                **Endfor**
              Append chromosome to pop_next_gen
        **Endfor**
      **Return** pop_next_gen
**Endfunction**

**Function generations (df, label, size, n_feat, n_parents, mutation_rate, n_gen, X_train, X_test, y_train, y_test)**
        best_chromo = Empty list
        best_score = Empty list
        population_nextgen = Call initilization_of_population with size and n_feat
        **For each generation** i **from** 1 **to** n_gen
                scores, pop_after_fit = Call fitness_score with population_nextgen, X_train, X_test, y_train, y_test
                Print the best score in generation i
                pop_after_sel = Call selection with pop_after_fit and n_parents
                pop_after_cross = Call crossover with pop_after_sel
                population_nextgen = Call mutation with pop_after_cross, mutation_rate, n_feat
                Append the best individual from generation i to best_chromo
                Append the best score from generation i to best_score
            **Endfor**
            **Return** best_chromo, best_score
      **Endfunction**

**Calling the function**
    Gene,score = generations (X, y, size = 80, n_feat = X.shape[1], n_parents = 64, mutation_rate = 0.20, n_gen = 5, X_train = X_train, X_test = X_test, y_train = y_train, y_test = y_test)

Finally, we present the main Algorithm 3.
**Algorithm 3** main**Input:** DataSpectre.xlsx
**Output:** Score, Modelchoice 
**Begin**
**Reading data:**
  data = Read Excel file: “Data.xlsx”
**Extracting features and labels:**
X = Feature selection without the target variable: “Class”
y = Reading data from the target variable: “Class”
**Splitting data into training and testing sets:**
X_train, X_test, y_train, y_test = Training and testing data split (X, y)
**Creating classifiers and models:**
Classifiers= [‘SVM’, ’Logistic’, ’RandomForest’, ’AdaBoost’, ’DecisionTree’, ’KNeighbors’, ‘XGBoost’]
models=[SVM(), LogisticRegression(), RandomForestClassifier(), AdaBoostClassifier(), DecisionTreeClassifier(), KNeighborsClassifier(), XGBClassifier()]
**Writing a function to train, test, and evaluate models:**
Score, Modelchoice **= Train_test_evaluate(**X_train, X_test, y_train, y_test, models**)**
                **G, S = Genetic(X, y, X_train, X_test, y_train, y_test,** Modelchoice)
                **Choose the indices associated with the highest scores in the variable ‘ind.’**
                **For vi in ind**
**for i, value in enumerate(G[vi])**
        **if value is True then**
                **append i to indices_true1**
                        **end**
**end**
**X_train_GA = X_train with columns selected using indices in indices_true1**
**X_test_GA = X_test with columns selected using indices in indices_true1**
Score, Modelchoice **= Train_test_evaluate(X_train_GA, X_test_GA**, y_train, y_test, models)
                **end**

### 3.5. Performance Metric

We will utilize the following evaluation metrics to gauge the performance of the models in our study [25,26]:

- Accuracy: This assesses the proportion of correctly predicted observations.
- Precision: This measures the proportion of accurate positive predictions.
- Mean Squared Error (MSE) calculates the average difference between predicted and actual values.
- Recall: This evaluates the proportion of actual positive observations that are correctly predicted.
- F1 Score: This is a weighted average of precision and recall.
- Matthews Correlation Coefficient (MCC): This correlation coefficient assesses the quality of binary and multiclass classifications.

These metrics are calculated from the following formulas:

(1) $$Accuracy=\frac{TP+TN}{TP+FP+TN+FN}$$

(2) $$Precison=\frac{TP}{TP+FP}$$

(3) $$Recall=\frac{TP}{TP+FN}$$

(4) $$F1\;score=2\times \frac{Precision\times Recall}{Precision+Recall}$$

(5) $$MCC=\frac{TP\times TN-FP\times FN}{\sqrt[{}]{\left(TP+FP\right)\left(TP+FN\right)\left(TN+FP\right)\left(TN+FN\right)}}$$

(6) $$MSE=\frac{1}{n}\sum_{i=1}^{n}{\left(Yi-\hat{Y}i\right)}^{2}$$

## 4. Results and Discussion

We will present our results and discussion, distinguishing the results of the analysis of multispectral measurements.

In this section, we will present the results obtained from classification using various classification algorithms and the classification results after feature optimization.

### 4.1. Spectral Measurements

The data in Table 1 highlight the performance of various algorithms in classification. A comprehensive analysis of key metrics reveals that the logistic regression algorithm performs superiorly in most metrics. These metrics include accuracy, precision, F-score, recall, and Matthews correlation coefficient (MCC). These results consolidate the strong position of logistic regression in this classification task.

The logistic regression algorithm obtains the highest accuracy rate of 82.60%. This indicates that it correctly predicts 82.60% of the samples. Additionally, its accuracy of 83.76% turns out to be the best among all algorithms, highlighting its low false positive prediction rate. Furthermore, an F-score of 82.33%, which skillfully combines precision and recall, confirms the balance between these two measures, reflecting a robust overall performance.

The Matthews Correlation Coefficient (MCC), reaching 74.71% for the logistic regression, demonstrates a strong agreement between the model predictions and the actual values. This reveals the considerable discrimination capacity of this model.

The SVM and Random Forest algorithms also perform well, with relatively high accuracy, precision, F-score, and recall rates. As a result, they represent viable alternatives.

In contrast, the Decision Tree algorithm performs worse in most metrics, with lower accuracy, precision, F-score, recall, and MCC scores. Adjustments or improvements may be necessary to increase its effectiveness in this classification task.

Table 2 produces the result produced by the genetic algorithm in five generations.

The genetic algorithm achieved its best score in the second generation with a score of 86.95%, but it struggled to maintain this level of performance in subsequent generations. It also achieved this score in the fifth generation, but intervening generations showed stagnation in performance. Adjustments may be required in the configuration of the genetic algorithm to consistently maintain or improve performance. Table 3 lists the specific wavelengths that led to higher scores across generations.

Based on the results obtained for the different wavelengths, we undertook classification operations according to the specific optimal generations. This approach allowed us to analyze the performances of the different generations with the wavelengths in more depth, thus contributing to a better understanding of the results.

Table 4 presents the classification metrics for different algorithms with the parameters of the lengths obtained during the second generation of the genetic algorithm. The key metrics included are accuracy, precision, mean square error (MSE), F-score, recall, and the Matthews correlation coefficient (MCC). The SVM shows an accuracy of 78.26%, which indicates that it correctly predicts about 78.26% of the samples. Its accuracy is also high at 79.42%. The F-score and recall are also solid, while the MCC is 68.10%. Overall, SVM gives good performance. Decision Tree has an accuracy of 65.21%, which is lower than most other algorithms. The precision and recall are also 65.21%. The F-score is 65.21%, reflecting a balance between precision and recall. However, the MCC is relatively low at 47.72%. Random forest achieves an accuracy of 69.56%, with a precision of 69.15%. F-score and recall are similar at 69.19%. The MCC is 54.41%. Random forest shows a reasonable performance. XGBoost has an accuracy of 78.26%, similar to SVM, with a precision of 79.08%. The F-score is slightly higher at 78.46%, as is the recall. The MCC is 67.52%, indicating good performance. KNeighbors performs similarly to Decision Tree, with an accuracy of 65.21%, a precision of 65.21%, an F-score of 65.21%, and an MCC of 47.72%. Logistic regression achieves the best accuracy at 82.60%, with an exceptional precision of 84.71%. The F-score is 82.22%, recall is 82.60%, and MCC is the highest at 75.31%. Logistics is the leading algorithm during this generation. AdaBoost performs worse than other algorithms, with an accuracy of 60.86%, a precision of 43.47%, an F-score of 49.27%, a recall of 60.86%, and an MCC of 51.63%.

In conclusion, the parameters selected during the second generation favor the performance of the Logistic regression and XGBoost algorithms, whether in terms of accuracy, precision, F-score, recall, or MCC.

Table 5 reveals the classification metrics for various algorithms using the wavelength parameters obtained in the fifth generation of the genetic algorithm. These metrics include accuracy, precision, mean square error (MSE), F-score, recall, and the Matthews correlation coefficient (MCC).

SVM maintains an accuracy of 73.91% and a precision of 75.36%. The F-score reached 74.38%, the recall remained at 73.91%, and the MCC stood at 60.85%. Although this performance has decreased slightly compared to the previous generation, it remains solid. Decision Tree maintains an accuracy of 65.21%, with a precision of 65.28%. The F-score stands at 65.09%, the recall reaches 65.21%, and the MCC stands at 47.86%. The performance remains similar to the previous generation. Random forest also presents a stable performance, with an accuracy of 73.91%, a precision of 74.12%, an F-score of 73.82%, a recall of 73.91%, and an MCC of 60.96%. XGBoost achieves an accuracy of 69.56%, with a precision of 70.14%. The F-score reaches 69.15%, the recall remains 69.56%, and the MCC is 54.88%. Although the performance has declined slightly, XGBoost remains a competent choice. KNeighbors maintains a similar performance to the previous generation, with an accuracy of 65.21%, a precision of 67.14%, an F-score of 65.94%, a recall of 65.21%, and an MCC of 47.71%. Logistic regression maintains a stable performance with an accuracy of 69.56%, a precision of 71.49%, an F-score of 70.29%, a recall of 69.56%, and an MCC of 54.28%.

However, AdaBoost exhibits a lower performance compared to other algorithms, with an accuracy of 60.86%, a precision of 43.47%, an F-score of 49.27%, a recall of 60.86%, and a MCC of 51.63%. To summarize, most algorithms experienced a performance loss during the fifth generation compared to the second generation. SVM and random forest maintain a solid performance. Decision Tree and KNeighbors perform similarly, while Logistic and XGBoost have regressed slightly. AdaBoost remains the algorithm with the least satisfactory performance.

### 4.2. Summary of Spectral Measurements

Table 6 summarizes spectral measurement accuracies for different algorithms, distinguishing between the complete data set, generation two, and generation five. Accuracy is a key indicator of a model’s ability to make accurate predictions.

SVM maintains a consistent accuracy of 79.42% between generation two and the entire dataset, but shows a slight drop to 75.36% at generation five. Decision Tree shows an improvement in accuracy from 60.86% at generation two to 65.21% at generation five. The accuracy of random forest is lower at generation two (69.15%), but it returns to 74.12% at generation five, which suggests an improvement over the previous generation. The accuracy sees a slight improvement at generation five. Logistic accuracy is high at generation two (84.71%) but drops significantly to 71.49% at generation five. AdaBoost’s accuracy remains low, showing no significant improvement between generations.

**Figure 8:**
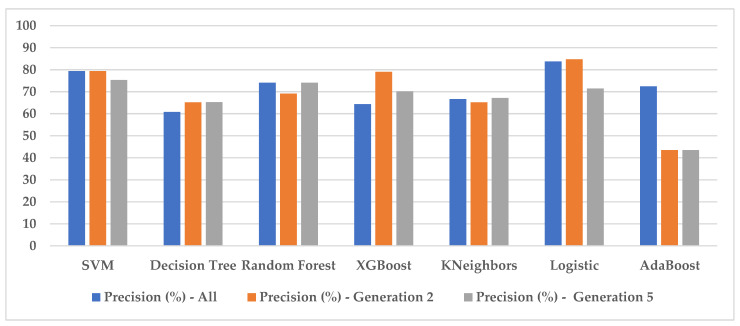
Histogram of the summary of spectral measurements.

These results highlight the variability in algorithm performance over different generations. Performance can fluctuate, increase, and sometimes decrease, underscoring the importance of monitoring model performance over time and adjusting algorithms accordingly to maintain quality results. Figure 8 shows the histogram of the synthesis.

These results show that the choice of generation and parameters plays an essential role in the performance of the algorithms. The improvement in accuracy between generations can be attributed to the better adaptation of parameters or features to a specific generation. On the other hand, variations in accuracy suggest that parameters optimized at one generation are only sometimes best for different generations. Users will need to consider these results to choose the algorithm and generation best suited to their classification task.

## 5. Conclusions

Our study examined the performance of different classification algorithms on spectral measurement data using a genetic algorithm for parameter optimization. The results reveal significant variations in algorithm accuracy between different generations, highlighting the importance of parameter optimization to achieve the best performance. The Logistic Regression, SVM, and random forest algorithms demonstrated performance stability, while XGBoost showed significant improvement in generation two but lost accuracy in generation five. AdaBoost was the algorithm that presented the least satisfactory performance across all generations. The use of deep learning in the context of spectral measurements could be possible, but certain considerations specific to this field could influence the choice of other, more traditional models. Deep learning often requires massive data sets to achieve optimal performance. If the spectral measurement dataset is relatively small, more data are needed to train a deep learning model effectively.

The quality improvement discussed in this study concerns the ability of our method to achieve a more precise classification of beans based on their spectral properties. This approach allows for a finer and more objective classification of beans, thereby reducing variations associated with manual sorting. Our method offers a technological alternative for agricultural research centers, offering a more objective, efficient, and precise solution for sorting cocoa beans. This could contribute to a significant improvement in the quality of the final products.

For future perspectives, this study could be extended by exploring more combinations of parameters and algorithms. Deep machine learning techniques or other emerging algorithms could also be considered. Furthermore, an in-depth analysis of the characteristics of spectral data and their interactions with optimization parameters could provide a better understanding of the performance of the algorithms. Finally, this research could find practical applications in various fields, including medical data classification, materials analysis, and other areas where spectroscopy is used for classification.

## Figures and Tables

**Table 1:** Results of classification metrics without parameter optimization.

Algorithms	Accuracy (%)	Precision (%)	MSE	F-Score (%)	Recall (%)	MCC (%)
SVM	78.26	79.42	0.6086	77.98	78.26	68.10
Decision Tree	60.86	60.86	1.3043	60.31	60.86	41.66
Random Forest	73.91	74.12	0.7826	73.82	73.91	60.96
XGBoost	65.21	64.34	1.13	64.18	65.21	48.27
KNeighbors	65.21	66.66	1.13	65.28	65.21	48.27
Logistic	82.60	83.76	0.69	82.33	82.60	74.71
AdaBoost	69.56	72.46	0.95	67.82	69.56	56.90

**Table 2:** Genetic algorithm results.

Generation	Score (%)
1	82.60
2	86.95
3	82.60
4	82.60
5	86.95

**Table 3:** The best wavelengths at the second and fifth generations.

Generation	2	5
Wavelength (nm)	410; 430; 490; 500; 510; 520; 530; 540; 550; 560; 580; 600; 610; 630; 690; 700; 710; 720; 740; 760.	380; 390; 400; 420; 430; 440; 450; 500; 510; 520; 550; 580; 590; 620; 630; 640; 660; 670; 680; 730.

**Table 4:** Results of generation 2 classification metrics.

Algorithms	Accuracy (%)	Precision (%)	MSE	F-Score (%)	Recall (%)	MCC (%)
SVM	78.26	79.42	0. 60	77.98	78.26	68.10
Decision Tree	65.21	65.21	1.13	65.21	65.21	47.72
Random Forest	69.56	69.15	0.95	69.19	69.56	54.41
XGBoost	78.26	79.08	0.60	78.46	78.26	67.52
KNeighbors	65.21	65.21	1.13	65.21	65.21	47.72
Logistic	82.60	84.71	0.56	82.22	82.60	75.31
AdaBoost	60.86	43.47	1.30	49.27	60.86	51.63

**Table 5:** Results of generation 5 classification metrics.

Algorithms	Accuracy (%)	Precision (%)	MSE	F-Score (%)	Recall (%)	MCC (%)
SVM	73.91	75.36	0.91	74.38	73.91	60.85
Decision Tree	65.21	65.28	1.13	65.09	65.21	47.86
Random Forest	73.91	74.12	0.78	73.82	73.91	60.96
XGBoost	69.56	70.14	0.95	69.15	69.56	54.88
KNeighbors	65.21	67.14	1.26	65.94	65.21	47.71
Logistic	69.56	71.49	1.08	70.29	69.56	54.28
AdaBoost	60.86	43.47	1.30	49.27	60.86	51.63

**Table 6:** Summary of spectral measurement accuracies.

Algorithms	Precision (%)–All	Precision (%)–Generation 2	Precision (%)–Generation 5
SVM	79.42	79.42	75.36
Decision Tree	60.86	65.21	65.28
Random Forest	74.12	69.15	74.12
XGBoost	64.34	79.08	70.14
KNeighbors	66.66	65.21	67.14
Logistic	83.76	84.71	71.49
AdaBoost	72.46	43.47	43.47

## Data Availability

The data is the property of the University and cannot be shared.
